# Digital Templating of Hip Arthroplasty Using Microsoft PowerPoint: A Pilot Study with Technical Details

**DOI:** 10.3390/bioengineering11040327

**Published:** 2024-03-28

**Authors:** Yonghan Cha, Jun Young Chung, Jin-Woo Kim, Jun-Il Yoo, Woohyun Lee, Jung-Taek Kim

**Affiliations:** 1 Department of Orthopaedic Surgery, Daejeon Eulji Medical Center, Eulji University School of Medicine, Daejeon 35233, Republic of Korea; 2 Department of Orthopaedic Surgery, Ajou University School of Medicine, Ajou Medical Center, Suwon 16499, Republic of Korea; 3 Department of Orthopaedic Surgery, Nowon Eulji Medical Center, Eulji University, Seoul 01830, Republic of Korea; 4 Department of Orthopedic Surgery, Inha University Hospital, Inha University College of Medicine, Incheon 22332, Republic of Korea; 5 Hallym University College of Medicine, Chuncheon 24252, Republic of Korea

**Keywords:** hip, arthroplasty, templating, digital, acetate

## Abstract

Templating is essential in hip arthroplasty preparation, facilitating implant size prediction and surgical rehearsal. It ensures the selection of suitable implants according to patient anatomy and disease, aiming to minimize post-operative complications. Various templating methods exist, including traditional acetate templating on both analog and digital images, alongside digital templating on digital images, which is categorized into 2D and 3D approaches. Despite the popularity of acetate templating on digital images, challenges such as the requirement for physical templates and result preservation persist. To address these limitations, digital templating with software like OrthoSize and Orthoview has been suggested, although not universally accessible. This technical note advocates for Microsoft PowerPoint as an effective alternative for 2D digital templating, highlighting its user-friendly features for image manipulation without needing specialized software. The described method involves scanning acetate templates, adjusting the images in PowerPoint 365 for size, position, and calibration on patient radiographs, and demonstrating reliability through preliminary assessments, with intraclass correlation coefficient (ICC) values indicating a high level of agreement for cup and stem size (ICC = 0.860, 0.841, respectively) but moderate for neck length (ICC = 0.592). We have introduced a method for performing 2D digital templating in the clinical field without the need for specialized software dedicated to digital templating. We believe this method significantly improves the accessibility to 2D digital templating, which was previously limited by the need for digital templating software. Additionally, it enables surgeons to easily establish arthroplasty plans and share them, overcoming the limitations of acetate templates.

## 1. Introduction

During the planning stages of surgery, various clinical tests are utilized, and one unique process in the planning of arthroplasty is templating [1,2]. Templating serves as an important first step in selecting the optimal implant from various types and sizes of devices that correspond with each individual patient’s anatomical variations and disease status to avoid difficult situations in the operating room [3,4]. Furthermore, templating extends beyond the simple sizing of implants. It involves rehearsing the entire surgery based on the patient’s image, identifying anticipated difficulties, and preparing the necessary instruments and implants from start to finish [5]. Templating plays a crucial role in preventing post-surgery complications such as dislocation and periprosthetic fracture by restoring hip length and offset and correcting patient-specific anatomical abnormalities [6].

Templating methods have evolved in response to changes in the medical environment, although the key concept of templating as rehearsing the entire surgery has not changed. Templating methods can be broadly divided into (1) acetate templating on analog hardcopy, (2) acetate templating on digital images, and (3) digital templating on digital images, which can be further subdivided into 2D and 3D templating [5,7,8].

With the widespread use of a picture archiving and communication system (PACS) in clinical settings, acetate templating on digital images has become popular. This method involves overlaying a fixed magnification ratio acetate template on a digital radiographic image, which can adjust the magnification ratio [9,10,11]. However, acetate templating on digital images still has several limitations. It requires acetate templates, which adds complexity for implant manufacturers and hospitals in terms of production and storage. Onscreen templating with acetate templates is volatile, making it difficult to save results, and the process of positioning and evaluating templates can be cumbersome.

To overcome these limitations, attempts have been made to implement digital templating [5,12]. Specialized software tools, such as OrthoSize (Zimmer Biomet, Warsaw, Indiana) and Orthoview (Materialize, UK), are required for digital templating [13,14,15,16,17]. Pongkunakorn et al. examined the accuracy of digital templating without the use of specialized software [18]. However, they utilized Adobe Photoshop (Adobe Systems, San Jose, CA, USA), a software specializing in image manipulation, to digitize scanned acetate templates and make them transparent for use on devices such as iPhones or MacBooks (Apple, Cupertino, CA, USA). This approach may not be easily accessible to surgeons who are unfamiliar with specialized programs or have difficulty accessing the Apple software ecosystem.

In our research, we have utilized Microsoft PowerPoint 365 (Microsoft Corporation, Redmond, WA, USA) for digital templating in clinical settings. PowerPoint is widely used for presentations and offers intuitive picture manipulation and utilization options. By using this software, the preparation of templates and templating itself can be performed with a single program, eliminating the need for multiple programs.

In this technical note, our objective was twofold: to furnish a comprehensive guide on executing the method and to present data comparing traditional acetate templating to 2D digital templating using Microsoft PowerPoint. Our hypothesis centered on the potential for digital templating with Microsoft PowerPoint to yield robust correlations with conventional acetate templating, in the prediction of cup and stem sizes, as well as femoral neck length, with a benchmark of excellence defined by an intraclass correlation coefficient exceeding 0.8. For quantitative comparison, a single rater undertook templating with both approaches on identical images of the hip radiograph. In this document, we have endeavored to provide comprehensive explanations regarding all the necessary operations for templating. However, we recommend utilizing the PowerPoint help function if any explanations are insufficient.

## 2. Materials and Methods

In templating using PowerPoint, two PowerPoint files are used. The first file, called the “Armamentarium” PowerPoint file, stores implant template images in a converted and ready-to-use format. Details regarding this format are elucidated in the subsequent subsection. Once organized, this file can be repeatedly used for multiple patients. The second file contains standardized hip anteroposterior (AP) radiographs and is referred to as the “Templating” PowerPoint file.

**Figure 1:**
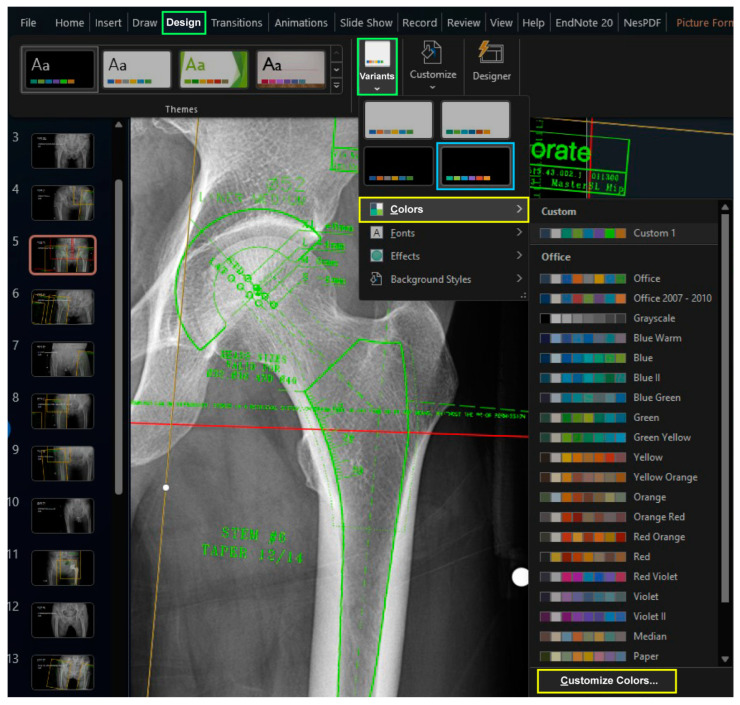
The design theme affects the background color and accent colors. Among the variants of the default theme (green boxes), a theme with a black background can be found (blue box). In the dropdown menu of variants (green box), one can customize the accent colors (yellow boxes).

When creating these two files, it is recommended to maintain an identical design theme to ensure consistent colors when copying an image from the “Armamentarium” PowerPoint file to the “Templating” PowerPoint file. It is recommended to select a variant with black background color from the default design themes in the “Design” Table Additionally, customizing the six accent colors is advised, as they will be used for the template images. To define the accent colors, select the “Customize Colors” command by clicking on the downward arrow under the “Colors” button after selecting the downward tab of “Variants” in the “Design” tab (Figure 1).

Exporting and importing design themes should ensure consistent design themes across both PowerPoint files. If the accent colors, defined by the design theme, differ between two files, copying and pasting template figures from one file to the other will result in figures appearing in different colors.

To adjust the position of the implant template figure overlaid on the radiograph in PowerPoint, basic techniques for adjusting the position of objects can be helpful:

- Zooming in/out: Hold down the “Ctrl” key and scroll the mouse wheel to zoom in or out.
- Temporarily override snap to grid or other objects: By default, PowerPoint’s automatic snapping feature is active to assist with positioning figures. However, automatic snapping can hinder the fine position adjustment during templating. To temporarily disable this feature, hold the “Alt” key while positioning the figure by dragging the mouse.
- Fine-tuning the placement of graphic objects: Typically, the position of a figure is adjusted by dragging it with the mouse, and rotation is achieved by using the object’s rotation handle. However, making fine adjustments to position and rotation using the mouse alone can be challenging. In such cases, with the figure selected, hold the “Ctrl” key and use the arrow keys to make small position adjustments. For fine rotations, hold “Ctrl” and “Alt” while using the left/right arrow keys.

### 2.1. Preparation of Digitally Scanned Images of the Acetate Template (“Armamentarium” PowerPoint File)

Scan all the acetate templates using a tabletop scanner and save them in JPEG format. Create a new PowerPoint file named “Armamentarium.” After adjusting the design theme, it is recommended that the slide size be expanded. This helps avoid repetitive resizing after copying the template images to the “Templating” PowerPoint file by containing and displaying the templates in the usually used size. We set the size of the “Armamentarium” PowerPoint file to a width of 100 cm and a height of 56.25 cm.

**Figure 2:**
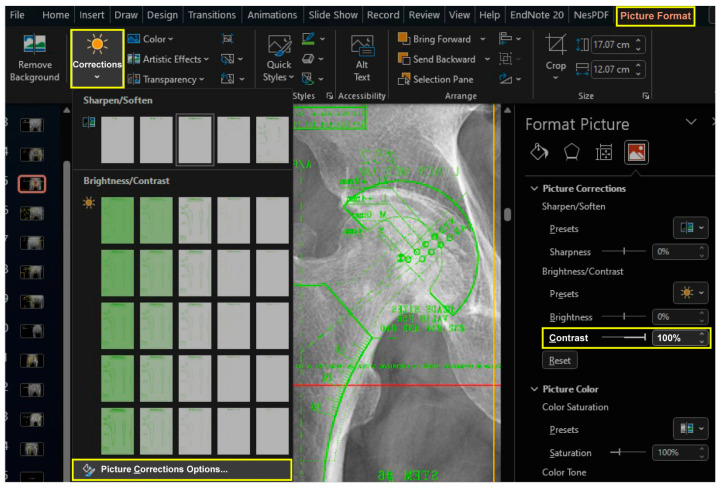
To enhance the differentiation between black and white, the contrast of template images was maximized. The “Picture Corrections” panel may be accessed via the “Picture Corrections Options” button located within the dropdown menu of the “Corrections” button located under the “Picture Format” tab (yellow boxes). It is important to note that this tab can only be accessed once a figure object has been selected.

Import the scanned images to the “Armamentarium” PowerPoint file. The “Picture Corrections” panel can be activated by clicking the “Picture Corrections Options” button in the dropdown menu of the “Corrections” button under the “Picture Format” tab, which is activated only after selecting a figure object. Increase the contrast of the images to +100% to clearly differentiate between black and white (Figure 2).

**Figure 3:**
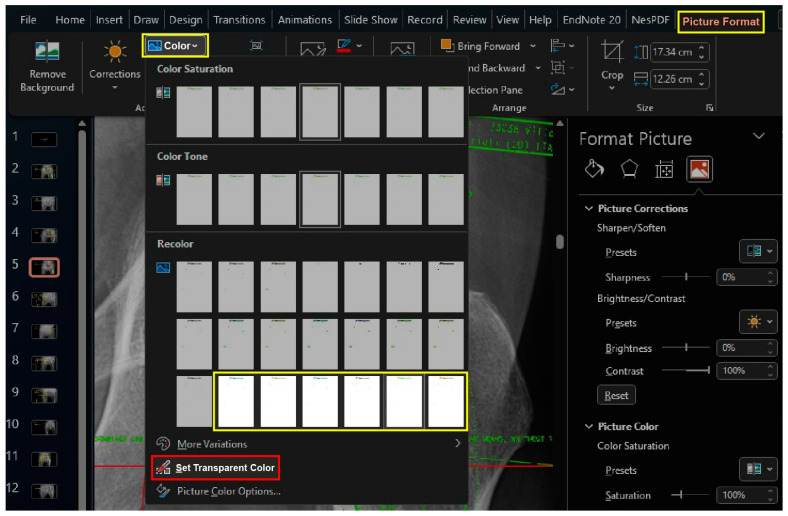
The lines within template images are capable of being altered through the utilization of the “Recolor” preset found on the dropdown menu located within the “Colors” button of the “Picture Format” Table The third row of the “Recolor” menu provides the option to change line colors to one of several predetermined accent colors. (yellow boxes). To remove the white background of a template image, access the “Picture Format” tab and click on the “Colors” button. From the dropdown menu, select “Set Transparent Color” and then click on the white background of the template image (red box).

While keeping the image selected, the lines of template images can be changed using the “Recolor” preset, which is found in the dropdown menu of the “Colors” button under the “Picture Format” Table The third row in the “Recolor” menu provides the option to change line colors to one of the predefined accent colors (Figure 3).

While keeping the image selected, click the “Set Transparent Color” button found in the dropdown menu of the “Colors” button under the “Picture Format” tab, and click the white background of the template image (Figure 3).

Activating the “Picture Border” feature, which outlines the border of the image, can aid in working with template images. Many digital templating software programs typically use light green to depict the contour of implants; however, based on the authors’ experience, using a color that is too prominent can obscure anatomical landmarks on the radiograph, making templating more challenging. Thus, the authors prefer the color “Fire brick” (R178, G34, B34) as it allows for clear visualization of both the implant contours and anatomical landmarks. However, for demonstration purposes and to enable readers to compare with other software programs, the examples provided in this note will use the color “Lawn Green” (R124, G252, B0), which is widely used in other software programs.

With the picture correction functions of Microsoft PowerPoint, the scanned images of acetate templates can be transformed into a transparent format that is ready to use. Once this “Armamentarium” PowerPoint file is saved, surgeons can initiate the templating process for their daily practice, commencing from the step detailed in the subsequent section.

### 2.2. Importing Images and Optimizing Implant Position (“Templating” PowerPoint File)

The radiograph is an essential element in the templating process as it provides information related to the patient’s anatomical variations. This step is crucial as errors at this stage can lead to larger errors in the subsequent templating process. Various literature has emphasized the importance of obtaining standardized radiographs [1,19]. One common error is the failure to obtain an AP view of the proximal femur due to not positioning the feet in a 15-degree internal rotation during image taking. According to standard imaging methods, there is an average magnification of approximately 20%, which can be influenced by various factors, making the use of magnification markers recommended [5,19,20]. As the standardized radiographic imaging methods go beyond the scope of this technical note, it is advisable to refer to other literature sources on this topic [1,19]. To export the standardized radiograph from the PACS, individuals should consult the help function of PACS viewers employed by the respective hospital. While importing it into the “Templating” PowerPoint file, accompanying brief patient identification information helps prevent mixing up patient data.

### 2.3. Calibration of Magnification between Radiograph and Template

**Figure 4:**
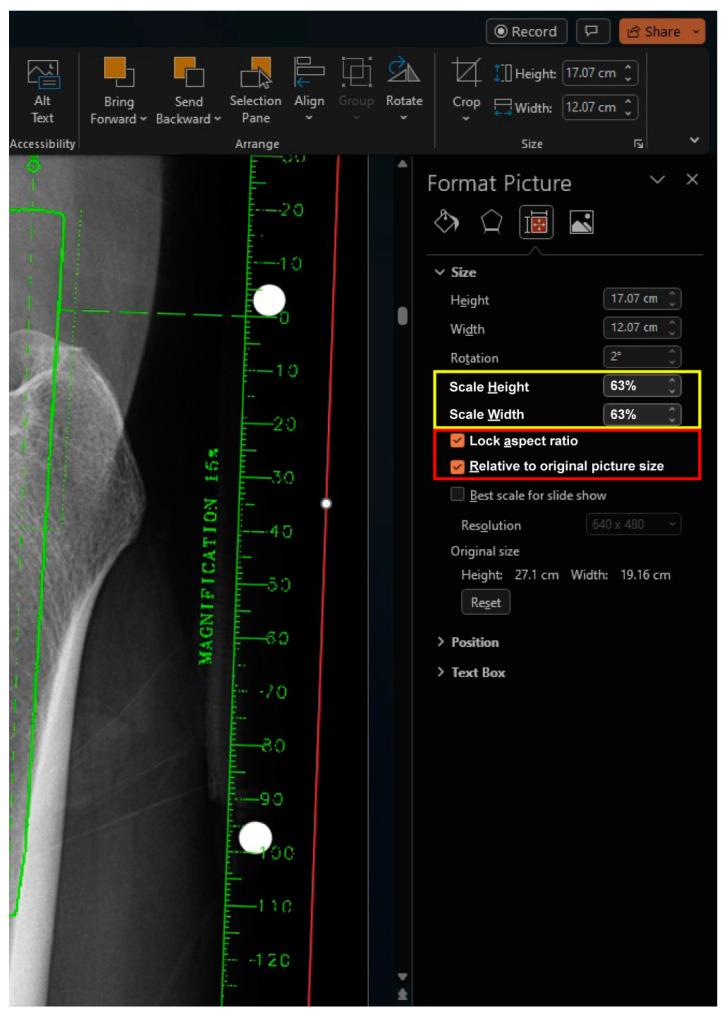
To match the magnification between the radiograph and the template, a known distance is matched to the ruler of the template by resizing the size of the template image. When using the mouse to drag the active frame, only the dragging points at each corner should be used. Avoid using the dragging points on each side of the frame. When utilizing the percentage of Scale Height or Scale Width in the size section, ensure that both the “Lock aspect ratio” and “Relative to original picture size” checkboxes are checked (red box). After the magnification factor has been established for a specific implant, it remains consistent and can be applied without repeating the calibration process. Thus, it is important to remember this factor throughout the templating process for the same implant (yellow box).

The magnification of the digital template is adjusted to match the marker of the radiograph (Figure 4). The ruler on the template is used to match the known size of the marker on the radiograph. While dragging the active frame with the mouse is an easy way to resize the image crudely, more precise adjustments can be made by toggling the percentage of Scale Height or Scale Width in the size section. This section can be accessed by selecting the size section arrow under the “Picture Format” Table When resizing the active frame using the mouse, it is important to avoid using the dragging points on each side of the frame and instead focus on using only the dragging points at each corner. When using the percentage of Scale Height or Scale Width in the size section, both the “Lock aspect ratio” and “Relative to original picture size” checkboxes should be checked, which is the default setting in the PowerPoint software. Once the magnification factor is determined for a specific implant, it remains constant within the same implant and can be applied without repeating the calibration process.

### 2.4. Optimization of Implant Position

**Figure 5:**
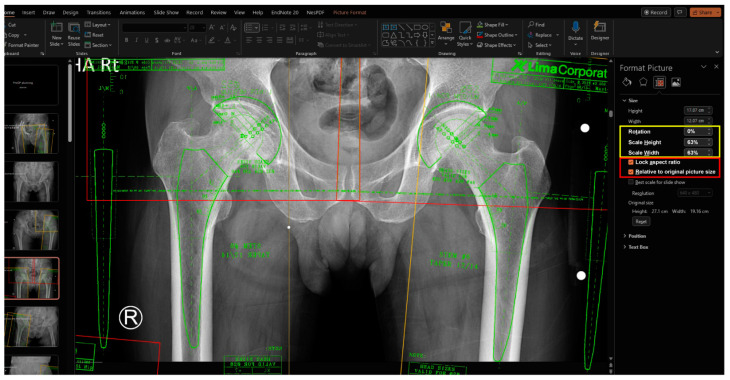
Once the magnification factor has been determined, it can be digitally inputted in the percentage input field of Scale Height or Scale Width in the size section when using a different size of the same implant. Additionally, the predetermined rotation angle can be typed in the input field of rotation. These input fields can be used for the fine adjustment of size and rotation as well (yellow box). When utilizing the percentage of Scale Height or Scale Width in the size section, ensure that both the “Lock aspect ratio” and “Relative to original picture size” checkboxes are checked (red box).

Microsoft PowerPoint provides various drawing functions, including lines and circles, which allow users to draw auxiliary lines on the radiograph. As this technique incorporates key concepts regarding the optimization of implant position, please refer to the literature for detailed explanations [10,19,21,22,23,24,25]. To apply these key concepts in PowerPoint, basic techniques for adjusting the position of objects are helpful. If a template of the implant in a different size is required, the user can copy it from the “Armamentarium” PowerPoint file and paste it onto the “Templating” PowerPoint file. The determined scale from the earlier calibration can be digitally inputted into the percentage input field of “Scale Height” or “Scale Width” in the “Size” section, which is activated by selecting the size section arrow of the “Picture Format” tab (Figure 5). Since the templates were scanned in the same environment, they have the same scale. Using the input field for rotation is an efficient way to avoid repetitive procedures as well.

### 2.5. Efficient Setup

To improve efficiency, dual monitors can be used to display the “Armamentarium” PowerPoint file and “Templating” PowerPoint file on separate screens, although there are no significant limitations to using a single monitor.

Enhanced accessibility can be achieved by utilizing pinned features in shortcuts, bookmarks, and recent files provided by Windows. Additionally, leveraging cloud services allows for greater flexibility and freedom from time and location constraints.

### 2.6. Agreement Analysis

The necessity to procure informed consent was exempted, as the investigation was based on radiographs that had already been obtained. The research protocol obtained approval from our hospital’s Institutional Review Board, given the concise nature of the comparison and utilization of preexisting radiographic data. In this preliminary assessment, a total of 20 preoperative radiographs taken for templating were included. From June to September 2023, we obtained 20 sets of bilateral hip anteroposterior (AP) views for patients scheduled for primary hip arthroplasty. We excluded images with metal fixation devices due to previous surgeries on the pelvis and femur from the preoperative imaging for templating.

We obtained 20 sets of bilateral hip anteroposterior radiographs for patients scheduled for primary hip arthroplasty, excluding images with metal fixation devices due to previous surgeries on either the pelvis or femur. The primary diagnoses for hip arthroplasty were osteonecrosis of the femoral head in nine patients, primary arthritis in two patients, dysplastic OA in two patients, post-traumatic OA in four patients, and femoral neck fracture in three patients. The mean age was 59.8 years. Nine patients were male, and 11 patients were female.

A rater executed the templating process utilizing both on-screen and 2D digital methodologies, with a one-month interval between the two implementations. The anticipated dimensions of the cup, stem, and neck length were documented and subjected to comparative analysis. The intraclass correlation coefficient (ICC) was employed to assess the agreement for each component [26,27].

## 3. Results

Cup sizes ranged from 46 to 58, while stem sizes ranged from 1 to 12.5. The calculated ICC values for cup, stem, and neck length were 0.860, 0.841, and 0.592, respectively.

## 4. Discussion

In this study, we have introduced a simple method for performing 2D digital templating in the clinical field, eliminating the need for specialized software previously deemed essential for digital templating. This method significantly enhances the accessibility of 2D digital templating, a crucial step forward given the real-world constraints imposed by the exclusive reliance on specialized templating software. Our method not only ease access to advanced templating techniques but also facilitates a more integrated and collaborative approach to surgical planning. By enabling surgeons to easily establish arthroplasty plans and share them, our approach addresses and effectively overcomes the limitations associated with traditional acetate templates. This advancement holds the promise of transforming preoperative planning processes, allowing for a more personalized, precise, and patient-centered approach to hip arthroplasty.

The method of acetate templating on digital images is widely used, especially with the widespread use of PACS in the clinical setting. This method involves overlaying the acetate template in a fixed magnification ratio on a digital radiographic image where the magnification ratio can be adjusted. However, this method using acetate templates poses certain challenges. It requires additional manufacturing and supply of acetate templates, posing complexity for implant manufacturers and hospitals in terms of storage. It is not easy for producers to supply templates for rarely used special implants, and users face difficulties in storing them. Moreover, acetate templates are usually limited in supply, restricting templating to specific locations where the acetate templates are stored. Furthermore, onscreen templating is not persistent. The results can only be observed when the acetate template is applied to the screen, and saving the results is challenging. Additionally, handling an acetabular template in one hand and finding and positioning a femoral template of an appropriate size can be cumbersome, and evaluating the neck length while holding both templates in an optimal position is not an easy task.

The limitations can be overcome with 2D digital templating. It eliminates the need for the additional physical space required to store the templates and reduces the burden of manufacturing and supplying acetate templates. Templating with PowerPoint can be performed anywhere a computer is available without limitations of supply. Two-dimensional digital templating does not impose acrobatic positions on users when positioning both acetabular and femoral templates simultaneously. The results of templating can be saved in a PowerPoint file or captured as a screenshot to share the results with the other participants of the operation or can be used for public or educational presentations.

Pongkunakorn et al. introduced the method of 2D digital templating without specialized software for templating [18]. However, their approach required several software tools within the Apple ecosystem. Considering that Microsoft Windows holds a dominant market share of 75% as the leading desktop computer operating system worldwide, it is likely that the majority of surgeons are more familiar with PowerPoint [28,29]. The method introduced in this note initially requires acetate templates only when scanning with a tabletop scanner. Once the scanned images of the acetate templates are collected, only the basic features of PowerPoint are needed to manage and view templates on patients’ radiographs. This approach allows surgeons familiar with the Windows environment to easily implement 2D digital templating. Once the library of templates is established, it becomes effortless to share and copy them without any loss of information. There are no limitations on the number of copies that can be made, making the process highly convenient.

A brief comparison of both methods indicated excellent agreements in cup and stem sizes and moderate neck length [26,30]. Since there is no direct comparison between digital and onscreen templating methods, the agreement between both approaches cannot be readily compared based on our results. The generally provided intraobserver reliability within each method ranges from 0.82 to 0.93 with cup, 0.86 to 0.95 with stem, and 0.56 to 0.71 with neck length, similar to our agreement of both methods [17,18]. To offer stronger evidence for the utility of digital templating using non-specialized software, additional research should be conducted to compare the templating prediction with actual implants used. This would help bridge the gap left by previous research primarily focused on using specialized digital templating software.

In this study, consistent with previous research, predictions of neck length exhibited lower agreement compared to those of cup and stem size. Typically, the ICC reported for cup measurements ranged from 0.82 to 0.93, for stem measurements from 0.86 to 0.95, and for neck length from 0.56 to 0.71 [17,18]. Our findings closely correspond with these ranges, with ICC values of 0.860 for cup size, 0.841 for stem size, and 0.592 for neck length. Predictions for the sizes of the cup and stem showed consistency as they are based on the fixed skeletal anatomy of the acetabulum and proximal femoral canal. However, predictions for neck length are not only dependent on the sizes of the cup and stem but also on the post-operative alignment changes from the preoperative positions of the pelvis and femur, leading to greater inconsistency. Research on templating methods may lead to a reduction in such inconsistency.

Based on the method we introduced here, the simplicity and accessibility of our method encourage its adoption across a broader spectrum of healthcare settings, potentially standardizing high-quality preoperative planning regardless of a facility’s resources. It invites future research into its application and efficacy across different types of arthroplasty and orthopedic procedures, suggesting a horizon of possibilities for its integration into surgical education and training programs. As we look ahead, we envision this method playing a pivotal role in enhancing the quality of care for patients requiring arthroplasty, by facilitating more accurate implant selections and alignment strategies, ultimately leading to improved surgical outcomes and patient satisfaction. We advocate for continued exploration and validation of this method, with a focus on its impact on post-operative outcomes, efficiency in surgical planning, and its role in the evolution of digital templating technologies.

## 5. Conclusions

In our research, we have developed and introduced a simple approach for executing 2D digital templating within clinical environments, eliminating the dependence on specialized, proprietary software traditionally required for such digital templating activities. This technique substantially increases the accessibility and utility of 2D digital templating, a domain previously hindered by the obligatory use of specific templating software. Furthermore, it grants surgeons the capability to efficiently formulate, refine, and disseminate comprehensive arthroplasty plans, thereby addressing and overcoming the physical limitations and inefficiencies associated with the use of conventional acetate templates in surgical planning.

## Data Availability

The datasets used and analyzed during the current study available from the corresponding author on reasonable request.
